# A151 RISK FACTORS FOR VENOUS THROMBOEMBOLISM AFTER HOSPITAL DISCHARGE IN PATIENTS WITH INFLAMMATORY BOWEL DISEASE: A SYSTEMATIC REVIEW AND META-ANALYSIS

**DOI:** 10.1093/jcag/gwab049.150

**Published:** 2022-02-21

**Authors:** M Gozdzik, D Unninayar, A Sarker, K Chin Koon Siw, J McCurdy

**Affiliations:** 1 University of Ottawa, Ottawa, ON, Canada; 2 Division of Gastroenterology, The Ottawa Hospital, Ottawa, ON, Canada

## Abstract

**Background:**

Inflammatory bowel disease (IBD) is a chronic inflammatory condition which is independently associated with venous thromboembolism (VTE). Although the absolute risk of VTE is greatest during hospitalization, the risk remains elevated in the early post-discharge period. The risk factors for VTE during this vulnerable period remain unknown.

**Aims:**

We performed a systematic review and meta-analysis to determine risk factors for VTE in the post discharge period among adult patients with IBD.

**Methods:**

We performed a systematic search of Embase, MEDLINE, and the Cochrane Central Register of Controlled Trials from inception through April 29, 2021 for publications that reported risk factors for VTE during the post-discharge period among patients with IBD. Study eligibility was assessed independently in duplicate without age or language restriction. We defined the post-discharge period as within 6 months of discharge. Pooled summary estimates of adjusted hazard/odds ratios, when available, were calculated for individual risk factors using random effects model with 95% confidence intervals. The analysis was performed when a minimum of three studies were available for a given risk factor. Heterogeneity was assessed using I^2^ statistic. Study quality was assessed using an adapted version of the National Institute of Health criteria.

**Results:**

We identified 10 studies from a total of 4339 abstracts that met our inclusion criteria: 8 population-based studies, 1 multicenter observational study and 1 single center observational study. Risk factors for post-discharge VTE were assessed at 6 weeks in 1 study, 1 month in 5 studies, 3 months in 2 studies, and 6 months in 2 studies. The variables assessed in our meta-analysis are reported in Table 1. Exposure to corticosteroid (odds ratio [OR], 1.77; 95% CI, 1.53–2.06) but not biologics (OR, 1.21; 95% CI 0.80–1.82) was associated with an increased risk of VTE. Furthermore, greater length of stay (OR 1.49; 95% CI, 1.01–2.20), ulcerative colitis (OR 1.41; 95% CI, 1.19–1.66), history of malignancy (OR 1.35; 95% CI, 1.12–1.62), and surgery during admission (OR 1.26; 95% CI, 1.12–1.42) but not female sex (OR 0.98; 95% CI, 0.88–1.10) or surgery type (OR 1.09; 95% CI, 0.75–1.57) were associated with increased risk of VTE after discharge. Overall, the study quality was rated as fair.

**Conclusions:**

In our meta-analysis, which consisted of moderate quality of evidence, we identified multiple risk factors associated with VTE in the post-discharge period. This work will help inform which factors should be considered for developing point of care clinical predictive models to help guide when extended VTE prophylaxis is required.

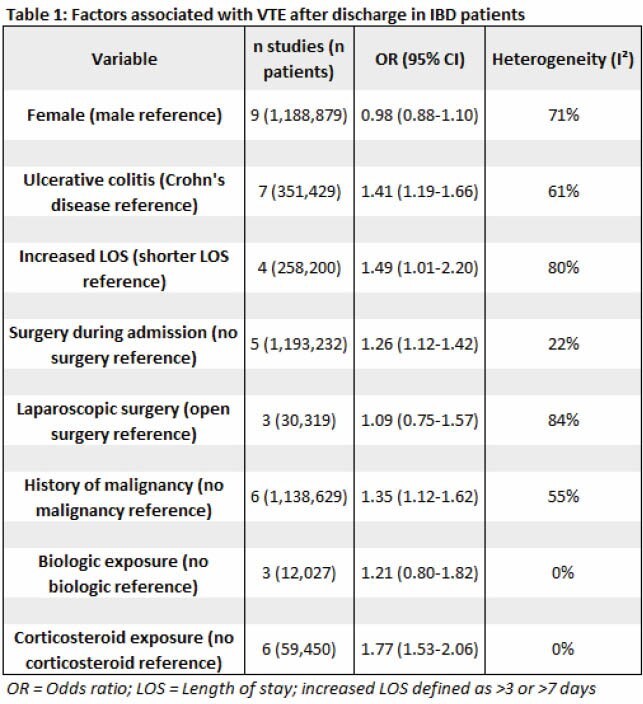

**Funding Agencies:**

None

